# Phenotypic and genetic dissection of seed shape related traits in soybean (*Glycine max* L.)

**DOI:** 10.3389/fpls.2026.1814388

**Published:** 2026-05-12

**Authors:** Kanglin Liu, Huan Yu, Sawaira Jadoon, Maoting Shen, Yajun Xiong, Yijie Chen, Zhiyu Liu, Junjun Liu, Bernard Gyebi-Nimako, Yu Zhang, Fan Zhang, Lijuan Qiu, Jun Wang

**Affiliations:** 1 The Shennong Laboratory/Institute of Crop Molecular Breeding, Henan Academy of Agricultural Sciences, Zhengzhou, China; 2 State Key Laboratory of Crop Gene Resources and Breeding, Institute of Crop Sciences, Chinese Academy of Agricultural Sciences, Beijing, China

**Keywords:** genetic dissection, genomic selection, QTL mapping, seed shape related traits, soybean, trait network

## Abstract

**Introduction:**

Seed shape and hundred-seed weight (HSW) are yield-related traits, but the interrelationships between them and a systematic genetic analysis are lacking.

**Methods:**

In this study, phenotypic evaluation of seed shape-related traits and HSW was performed in a recombinant inbred line (RIL) population derived from the cross between “ZD41” and “ZYD02878.” All seed shape-related traits (SW, seed width; SL, seed length; ST, seed thickness; SP, seed perimeter; SA, seed area; SLW, seed length-to-width ratio; SLT, seed length-to-thickness ratio; SWT, seed width to thickness ratio) were evaluated. Broad-sense heritability was calculated. Correlation analysis, path analysis, QTL mapping (using ICIM, EMMAX, and TASSEL), and genomic selection (using rrBLUP) were performed.

**Results:**

All seed shape-related traits exhibited approximately normal distributions in the RIL population. The broad-sense heritability of seed shape-related traits ranged from 0.67 to 0.90, indicating that additive genes played a major role in expressing all studied traits. Correlation analysis revealed that SW showed the highest positive correlation with HSW (r = 0.86–0.87, P < 0.001) among the three measured seed shape-related traits (SL, SW, and ST), which agrees with the finding that SW was the only trait showing a positive direct effect on HSW in path analysis. A total of 98 QTL corresponding to 59 unique regions were identified by ICIM, EMMAX, and TASSEL. Three genes, *Glyma.02G255900, Glyma.02G270100*, and *Glyma.02G275200*, were identified as candidates for seed shape regulation. Genomic selection for seed shape-related traits using rrBLUP revealed that the prediction accuracy of SL, SW, ST, SP, and SA reached 0.65, 0.68, 0.65, 0.67, and 0.68, respectively, based on genome-wide SNP markers.

**Discussion:**

This study provided a systematic dissection of seed shape-related traits in terms of both phenotypic and genetic aspects, which laid a solid foundation for gene cloning and could be used in breeding programs in the future.

## 1 Introduction

Soybean is a globally significant source of plant-based protein and vegetable oil (Qin et al., 2022; Zhang et al., 2022; Fang et al., 2017; Hu et al., 2023b). In 2024, China imported more than 100 million tons of soybeans in order to meet the domestic need of the country (https://www.stats.gov.cn/sj/). Increasing domestic soybean yield represents a critical strategy for reducing this reliance on imports (Liu et al., 2025). Hundred-seed weight is a key agronomic trait determining crop yield (Vogel et al., 2021; Hu et al., 2023b). Seed shape-related traits, including seed length (SL), seed width (SW), and seed thickness (ST), are major factors influencing both hundred-seed weight (HSW) and appearance quality in soybean (Liang et al., 2005; Kumawat and Xu, 2021).

To date, over 300 QTL associated with seed shape have been reported by various researchers (www.soybase.org). Recombinant inbred lines (RILs) and chromosome segment substitution lines (CSSLs) are commonly used as mapping populations for quantitative trait loci (QTL) mapping of soybean seed size (Cheng et al., 2016; Hina et al., 2020; Kumar et al., 2023; Liang et al., 2008; Luo et al., 2023; Teng et al., 2018, 2019; Kumawat and Xu, 2021; Moongkanna et al., 2011; Salas et al., 2006; Yang et al., 2017; Zhang et al., 2025). Using those populations, two pleiotropic QTL associated with SL, S21–S22 and O23–O19/O19–O21, have been fine-mapped (Niu et al., 2013a). Analysis of a natural population comprising 257 accessions from six geographical ecoregions in China identified 59 QTL exhibiting genotype-by-environment interactions (Niu et al., 2013b). Linkage analysis in an RIL population of 184 lines detected 12 QTL, of which five were related to seed shape, while association mapping in 219 cultivated soybean accessions identified 41 significant SNP loci, of which seven were related to seed shape (Hu et al., 2013). Using RIL and sub-F_2_ populations across three environments, a major seed shape QTL (*qSS3*) was mapped, and the effects were validated in NIL populations (Cui et al., 2020). Integrating findings from multiple studies, a meta-analysis localized 23 consensus QTL onto 17 linkage groups, and 37 candidate genes were designated (Gao et al., 2019).

To date, at least 17 genes have been identified that directly regulate soybean seed traits, including SL, SW, ST, and SP (Duan et al., 2023). Knocking out *GmGA3ox1* suppressed gibberellin (GA) biosynthesis in soybean, leading to significantly reduced seed length without affecting seed thickness and width (Hu et al., 2022). *GmPDAT*, involved in acyl lipid metabolism, was reported to regulate oil deposition as well as seed length and thickness (Liu et al., 2020). Genes that positively regulate SL and SW include *GmST05*, *GmUSPL1*, and *GmJAZ3*, while *Dt2* acts as a negative regulator of both SL and SW (Duan et al., 2022; Liang et al., 2022; Hu et al., 2023a; Yuan et al., 2024). Overexpression of *GmMFT* and *GmSW17* resulted in significant increases in seed length, width, and thickness (Cai et al., 2023; Liang et al., 2024). Conversely, plants overexpressing *SW16.1* exhibited a significant decrease in SL, SW, and ST (Chen et al., 2023a). Furthermore, *Gmdtm1–1* and *Gmdtm 1–2* had smaller seed area and perimeter than Williams 82 due to *GmNAP1* mutations (Tang et al., 2020).

Genomic selection (GS), initially proposed for animal breeding, has since been extensively studied in crop science (Meuwissen et al., 2001). The application of GS to complex traits may enable a faster rate of genetic improvement over time (Ravelombola et al., 2021). The factors influencing GS prediction accuracy include trait heritability, marker number and density, genetic relationship between the training population and the breeding population, training population size, and modeling methods (Alemu et al., 2024). GS has been widely applied in soybean for traits such as yield, plant height, seed weight, protein and oil content, as well as amino acid composition (Ma et al., 2018; Qin et al., 2019; Bandillo et al., 2023; Van der Laan et al., 2025). However, scarce studies have been conducted on GS for seed shape-related traits, which need to be investigated.

To address these questions, in this study, QTL identification related to seed shape-related traits was performed using both linkage and association analyses by phenotyping across two distinct environments over a RIL population derived from the cross between large-seeded cultivated soybean cultivar “ZD41” and the small-seeded wild soybean “ZYD02878,” and then candidate genes were screened based on SNP variation, haplotype analysis, and expression analysis with the purpose of delving into the relationship between seed shape-related traits and hundred-seed weight. This study provided a solid genetic basis for the molecular cloning of genes regulating seed shape.

## 2 Materials and methods

### 2.1 Plant materials

The RIL population, consisting of 364 lines, was developed previously and described (Chen et al., 2023b). Basically, the maternal and paternal parents of the RIL are ZD41 and ZYD02878, with significant difference in seed size. The RIL-F_8_ and F_9_ were planted at Yangtze University (30.37°N, 112.06°E) and A’jian Farm (34.41°N, 115.98°E) in the summers of 2020 and 2021, respectively, and hereafter were designated as 20JZ and 21SQ. The spaced planting design with uniform 1-meter row spacing was used (Wang et al., 2024). The NAM population, constructed from crosses between ZD41 and 35 paternal parents, was planted in 20JZ for candidate gene haplotype analysis (Song et al., 2024).

### 2.2 Phenotyping of seed shape related traits

Matured seeds from one randomly selected plant per line from both the RIL and NAM populations were threshed. Seed shape data—including measured traits, namely seed area (SA, mm²), seed perimeter (SP, mm), seed length (SL, mm), seed width (SW, mm), seed thickness (ST, mm), as well as derived traits, namely seed length-to-width (SLW), seed length-to-thickness (SLT), and seed width-to-thickness (SWT), were collected using a seed shape scanner (SC-G, Hangzhou Wanshen Detection Technology Co., Ltd., China) (Ren et al., 2021). Generally, the seeds on the plate were scanned to capture a two-dimensional image, in which the thinnest dimension (seed thickness) of the seeds was placed vertically; then, the length, width, and area were calculated using models, and the thickness of the seeds was obtained by determining the width of seeds fixed by two large combs, in which the seeds were placed linearly between comb teeth and the thinnest dimension was placed horizontally. Hundred-seed weight (HSW, g) was measured using a 0.01 g precision balance.

### 2.3 Statistical analysis

First, the 3-sigma principle was applied to remove outliers using R code (Khan et al., 2021). Descriptive statistical analysis of seed shape-related traits was then performed using R packages such as Hmisc (Harrell, 2003). Transgressive segregation was defined as the phenotype value of RIL population individuals being higher or lower than that of the parents. Correlation analysis was conducted using functions such as Pearson within the Hmisc (5.2-3) R package (Khan et al., 2021). Principal Component Analysis (PCA) was carried out using factoMineR (2.11) and factoextra (1.0.7) (Irnawati et al., 2021). Path analysis was implemented using the sem (3.1-16), lavaan (0.6-19), and agricolae (1.3-7) R packages, employing the average values of HSW and other seed shape-related traits across two environments, with HSW as the dependent variable (Y) and the other seed shape-related traits as independent variables. Total effect, direct effect, and indirect effect were used to characterize path coefficients, and the top three total effects among all traits were plotted in a network using visNetwork (Fox, 2006; Rosseel, 2012; De Mendiburu, 2019). Based on Pearson correlation analysis, Plant Trait Networks (PTN) were constructed among different traits with default parameters of r = 0.2 and P = 0.05 using the MultiTraits R package, and the network is characterized by the metrics of degree (K), closeness (C), betweenness (B), and clustering coefficient (CC) (He et al., 2020b; Li et al., 2022).

The variance among the RIL population and across different environments was estimated by two-way ANOVA (Nandigam et al., 2025). The broad-sense heritability (h²) of each seed shape-related trait was calculated using the following formula:


$${\text{h}}^{2}=\frac{{\text{V}}_{\text{g}}}{{\text{V}}_{\text{g}}+\frac{{\text{V}}_{\text{gl}}}{\text{L}}+\frac{{\text{V}}_{\text{e}}}{\text{PL}}}$$

where Vg represents the genotypic variance, Vgl denotes the genotype-by-environment interaction variance, Ve is the error variance, L indicates the number of environments, and P signifies the number of replications. Broad-sense heritability calculations were implemented using a completely randomized model via the lme4 (1.1-35.5) package (Bates et al., 2015).

### 2.4 Genotyping and QTL mapping

This RIL population was planted at the College of Agriculture, Yangtze University, in 2022. Young leaves were collected at the V5 stage for genomic DNA extraction. DNA was extracted using the CTAB method (Doyle and Doyle, 1990). Genotyping was conducted using a 200K SNP chip by Beijing Compass Biotechnology Co., Ltd (Song et al., 2024). The raw genotype data were then sorted, filtered, and imputed using PLINK (Purcell et al., 2007), TASSEL, and Beagle software, respectively, resulting in 85,313 high-quality SNPs (minor allele frequency <0.05, site completeness >0.8). Based on the high-density genetic map, ICIM analysis was performed using the BIP module in QTL IciMapping software to execute ICIM-ADD, with the LOD threshold set to 2.5, a scanning step of 1 cM, and other parameters set to default (Meng et al., 2015). Based on the Mixed Linear Model (MLM), the kinship matrix was calculated using emmax-kin-inter64 in EMMAX as a covariate; the top five principal components (PCs) were computed using GCTA and incorporated as covariates along with the kinship matrix into the association study using TASSEL. The significance threshold for both was set at −log_10_(1/m) (m = 85,313). Significant loci were selected, with flanking 100 kb regions defined as candidate intervals, and Manhattan and QQ plots were generated using the R package CMplot (Chen et al., 2023b).

### 2.5 Candidate gene screening

Using the Wm82.a2.v1 genome as a reference, LD analysis of association regions was performed with the R package IntAssoPlot (He et al., 2020a). The target interval was narrowed based on r² (>0.8), and genes containing four mutation types—nonsynonymous, splicing, stopgain, and stoploss—were further screened through single nucleotide polymorphism (SNP) variation analysis. For potential candidate genes, expression profile datasets from the public soybean transcriptome database PPRD were used to analyze gene expression levels in different tissues (root, stem, leaf, seed). Expression data were normalized and visualized as heatmaps using the R package pheatmap. Haplotype analysis was conducted using the HaploAssistant (Yu et al., 2024). Haplotypes with fewer than 10 genotypes were excluded. The significance of phenotypic differences between haplotypes was tested using the Wilcoxon rank-sum test. Combining candidate gene expression profiles and haplotype analysis results, candidate genes were functionally annotated based on the SoyBase database to identify genes regulating seed development and shape formation.

### 2.6 Genomic selection

To evaluate the impact of different marker sets on prediction accuracy in genomic selection (GS) for seed shape-related traits, we constructed three marker sets: marker set 1 (M1, 85,313 genome-wide SNPs), marker set 2 (M2, 5,101 SNPs linked to seed shape-related QTL), and marker set 3 (M3, 80,212 genome-wide SNPs excluding trait-associated SNPs). Models were evaluated by 5-fold cross-validation for 20 iterations. All marker sets were treated as random effects in rrBLUP (Endelman, 2011).

## 3 Results

### 3.1 Phenotypic analysis of seed shape-related traits

**Figure 1:**
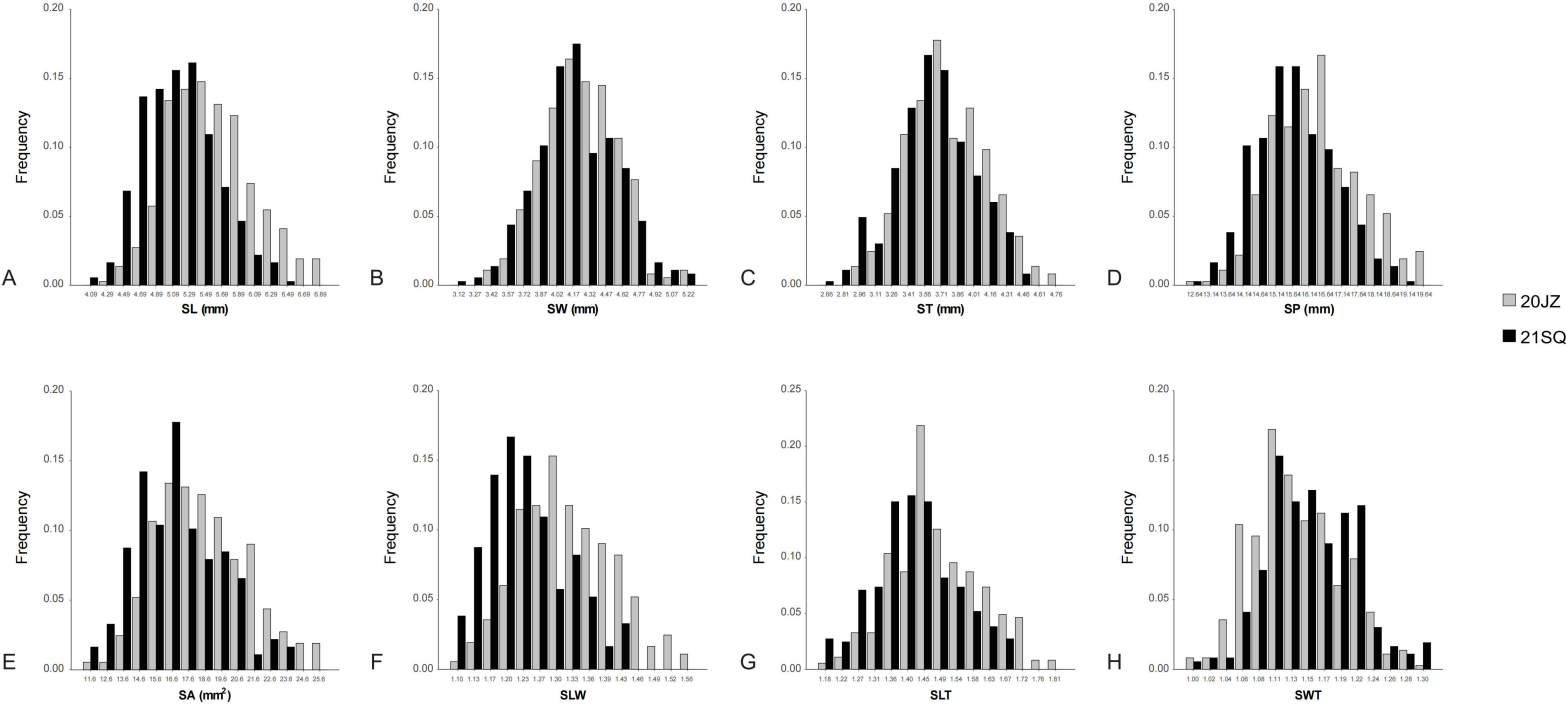
Frequency distribution of seed shape-related traits. **(A–H)** represent the frequency distribution of SA, SP, SL, SW, ST, SLW, SLT, and SWT, respectively, under two environments (20JZ and 21SQ). SA, seed area; SP, seed perimeter; SL, seed length; SW, seed width; SLT, seed length to seed thickness; SWT, seed width to seed thickness.

The 364 RILs used in this study were planted in two environments (20JZ and 21SQ) and phenotyped for seed shape-related traits. After removing outliers, the number of valid measurements for seed shape-related traits ranged from 337 to 364. The results showed that “ZD41” had significantly larger mean values for SL (7.32–7.64 vs 3.34–3.97), SW (6.23–6.47 vs 2.73–3.02), ST (5.98–6.16 vs 2.08–2.35), SA (38.30–38.60 vs 7.12–9.13), and SP (22.73–23.65 vs 10.15–11.67) than “ZYD02878.” In contrast, “ZD41” exhibited lower values for key shape descriptors—specifically, the SLW (1.17–1.18 vs 1.22–1.31), SLT (1.22–1.24 vs 1.61–1.69), and SWT (1.04–1.05 vs 1.29–1.31) (**Table 1**). These findings collectively demonstrated that “ZD41” produces seeds that are both larger and more spherical than those of the wild soybean “ZYD02878.” All seed shape-related traits of the RIL population in 20JZ and 21SQ approximately followed a normal distribution (**Figure 1**). Except for ST in 21SQ, all traits showed skewness >0.00 and kurtosis <0.00, exhibiting platykurtic and right-skewed distributions (**Table 1**; **Figures 1A–H**). For the parental lines (ZYD02878 and ZD41), the range of all measured traits (excluding SWT) were broader in the 20JZ than in 21SQ. Likewise, in the RIL population, 20JZ displayed broader ranges and higher mean values for every trait except SWT compared with 21SQ. Transgressive segregation was observed in the derived traits, e.g., SLT, SLW, and SWT, but not in measured traits (SL, SW, ST, SP, and SA) (**Table 1**). One-way ANOVA revealed that >70% of the total variance observed in each trait (SA (84%), SP (83%), SL (73%), SW (90%),ST(86%), SLW (72%), SLT (77%), SWT (72%)) was attributable to variance among genotypes, suggesting that the variation observed was mostly genetic rather than environmental (**Appendix A**). Similarly, SW exhibited the highest heritability (0.90), followed by SA (0.89), SP (0.88), ST (0.87), SL (0.83), SLW (0.81), and SLT (0.75), with SWT showing the lowest (0.67) (**Table 1**). This suggests that the expression of these traits is stable across different environments and is predominantly influenced by genetic variation.

**Table 1:** Descriptive statistics and broad-sense heritability of seed shape-related traits.

Trait	Env.	P1	P2	Max	Min	Mean	Sd	Skenes	Kurtosis	P Value	h^2^
SL (mm)	20JZ	7.64	3.97	6.86	4.14	5.49	0.51	0.29	−0.23	0.00	0.83
	21SQ	7.32	3.34	6.30	3.99	5.06	0.45	0.32	−0.28	0.00	
SW (mm)	20JZ	6.47	3.02	5.14	3.34	4.19	0.34	0.03	−0.28	0.32	0.90
	21SQ	6.23	2.73	5.12	3.12	4.11	0.37	0.19	−0.17	0.14	
ST (mm)	20JZ	6.16	2.35	4.74	2.83	3.73	0.37	0.18	−0.33	0.14	0.87
	21SQ	5.98	2.08	4.46	2.66	3.56	0.35	-0.02	−0.31	0.23	
SP (mm)	20JZ	23.65	11.67	19.80	12.47	16.23	1.33	0.28	−0.22	0.00	0.88
	21SQ	22.70	10.15	18.75	12.39	15.38	1.22	0.29	−0.33	0.02	
SA (mm2)	20JZ	38.60	9.43	25.62	10.63	18.11	2.84	0.33	−0.26	0.00	0.89
	21SQ	38.30	7.12	23.46	10.62	16.35	2.61	0.39	−0.26	0.00	
SLW	20JZ	1.18	1.31	1.56	1.10	1.31	0.09	0.25	−0.45	0.00	0.81
	21SQ	1.17	1.22	1.43	1.07	1.22	0.08	0.59	−0.31	0.00	
SLT	20JZ	1.24	1.69	1.80	1.18	1.47	0.12	0.22	−0.27	0.02	0.75
	21SQ	1.22	1.61	1.67	1.12	1.40	0.11	0.17	−0.27	0.02	
SWT	20JZ	1.05	1.29	1.29	0.98	1.13	0.06	0.31	−0.36	0.00	0.67
	21SQ	1.04	1.31	1.30	1.00	1.14	0.06	0.21	−0.13	0.04	

SA, seed area; SP, seed perimeter; SL, seed length; SW, seed width; SLT, seed length to thickness; SWT, seed width to thickness; SLW, seed length to width.

### 3.2 Interrelationships between HSW and seed shape related traits

**Figure 2:**
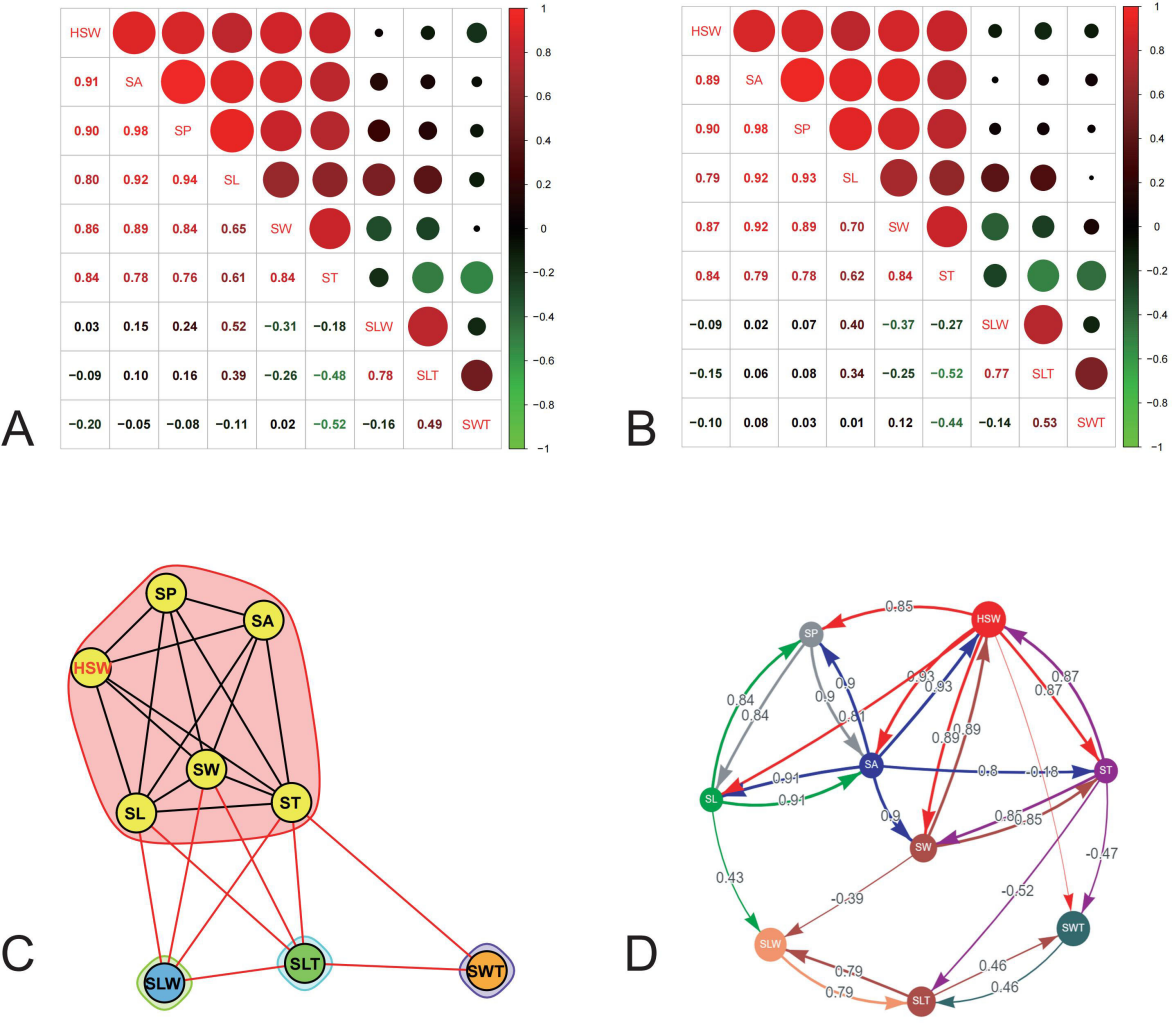
The correlation between HSW and seed shape-related traits. **(A, B)** Correlation heatmap of seed shape-related traits and HSW in 20JZ and 21SQ. **(C)** Plant trait network of seed shape-related traits and HSW, which was generated using the MultiTraits R package. **(D)** Path analysis of seed shape-related traits and HSW. Top three total effects of each target trait were displayed in network using visNetwork. SA, seed area; SP, seed perimeter; SL, seed length; SW, seed width; SLT, seed length to thickness; SWT, seed width to thickness; SLW, seed length to width.

Pearson correlation coefficients among different seed-related traits revealed that HSW, SA, SP, SL, SW, and ST exhibited significant positive correlations (r = 0.65–0.98, *P* < 0.001) across two environments. Among these, SA and SP consistently showed the strongest correlation (r = 0.98, P < 0.001). HSW exhibited strong positive correlations (r > 0.79, P <0.001) with the directly measured traits, specifically SA, SP, SW, ST, and SL, in descending order (**Figures 2A, B**). In contrast, the traits weakly associated with HSW were the derived traits SLW, SLT, and SWT.

Based on the correlation analysis, a plant trait network was constructed. The results indicated that ST, SL, and SW possessed the three highest values for degree (*K*), closeness (*C*), and betweenness centrality (*B*), identifying them as the central hubs within the network (**Figure 2C**; **Appendix B**). Within this network, HSW was directly connected to all five measured traits (SL, SW, ST, SP, and SA) but not to the derived traits (SLT, SWT, and SLW). This network architecture is consistent with the results of the correlation analysis.

Path analysis revealed that all five measured seed shape traits had positive total effects on HSW, ranging from 0.81 to 0.93. Among these, only SW exhibited a positive direct effect, while the other traits influenced HSW indirectly (**Figure 2D**; **Appendix C**). In contrast, all derived traits showed negative total effects on HSW.

### 3.3 QTL identification for seed shape related traits

**Figure 3:**
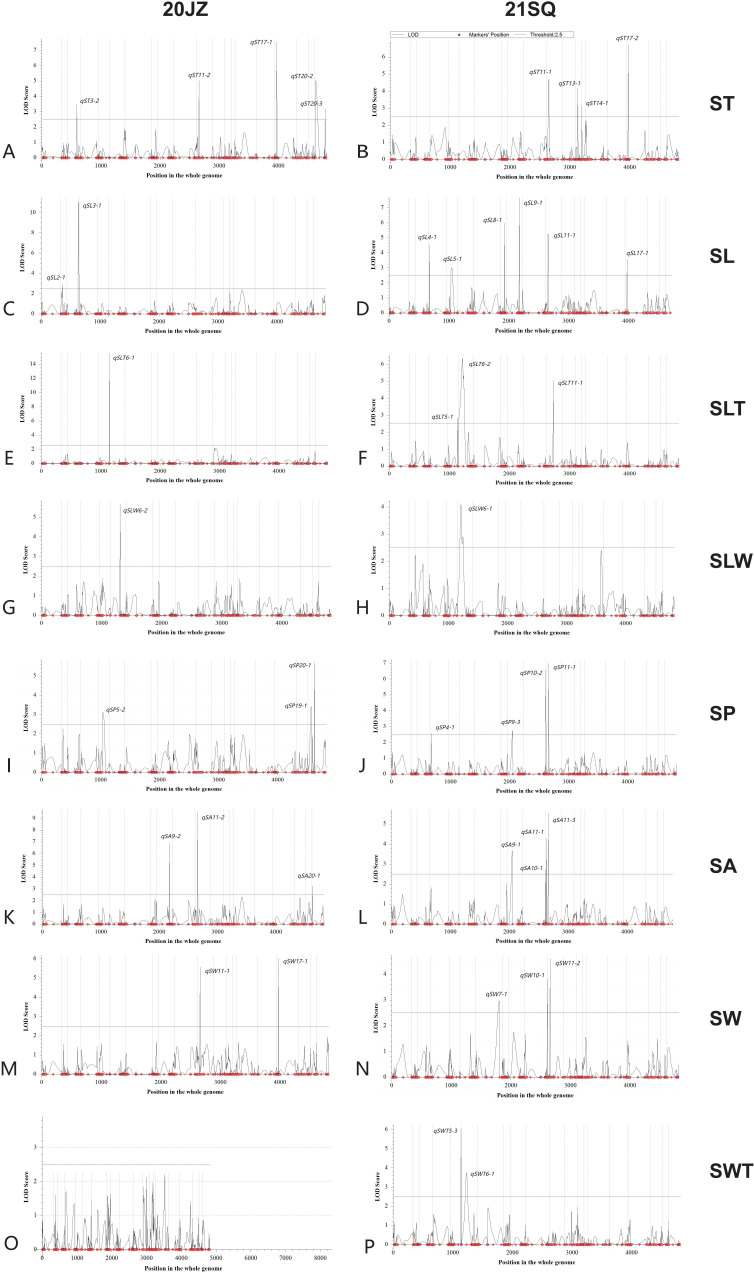
Seed traits related QTL mapped for **(A, B)** ST, seed thickness; **(C, D)** SL, seed length; **(E, F)** SLT, seed length to seed thickness; **(G, H)** SLW, seed length to width; **(I, J)** SP, seed perimeter; **(K, L)** SA, seed area; **(M, N)** SW, seed width; **(O, P)** SWT, seed width to seed thickness by ICIM in two environments (20JZ and 21SQ).

**Figure 4:**
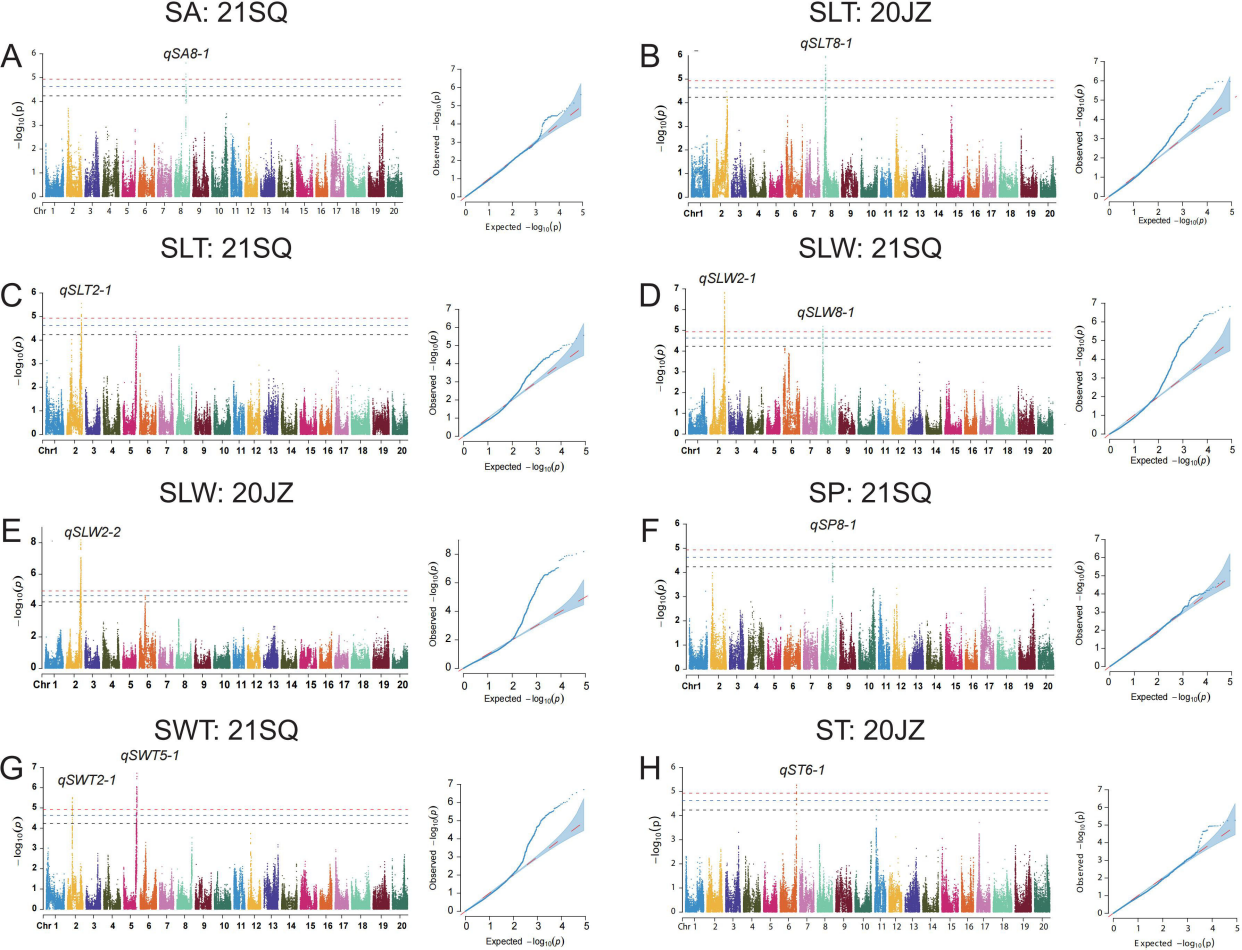
Manhattan plots and quantile-quantile (QQ) plots of seed shape related traits using EMMAX in two environments (20JZ and 21SQ), including **(A)** *qSA8-1*; **(B)** *qSLT8-1*; **(C)** *qSLT2-1*; **(D)** *qSLW2-1* and *qSLW8-1*; **(E)** *qSLW2-2*; **(F)** *qSP8-1*; **(G)** *qSWT2-1* and *qSWT5-1*; **(H)** *qST6-1.SA*, seed area; SP, seed perimeter; ST, seed thickness; SLW, seed length to width; SLT, seed length to seed thickness; SWT, seed width to seed thickness.

**Figure 5:**
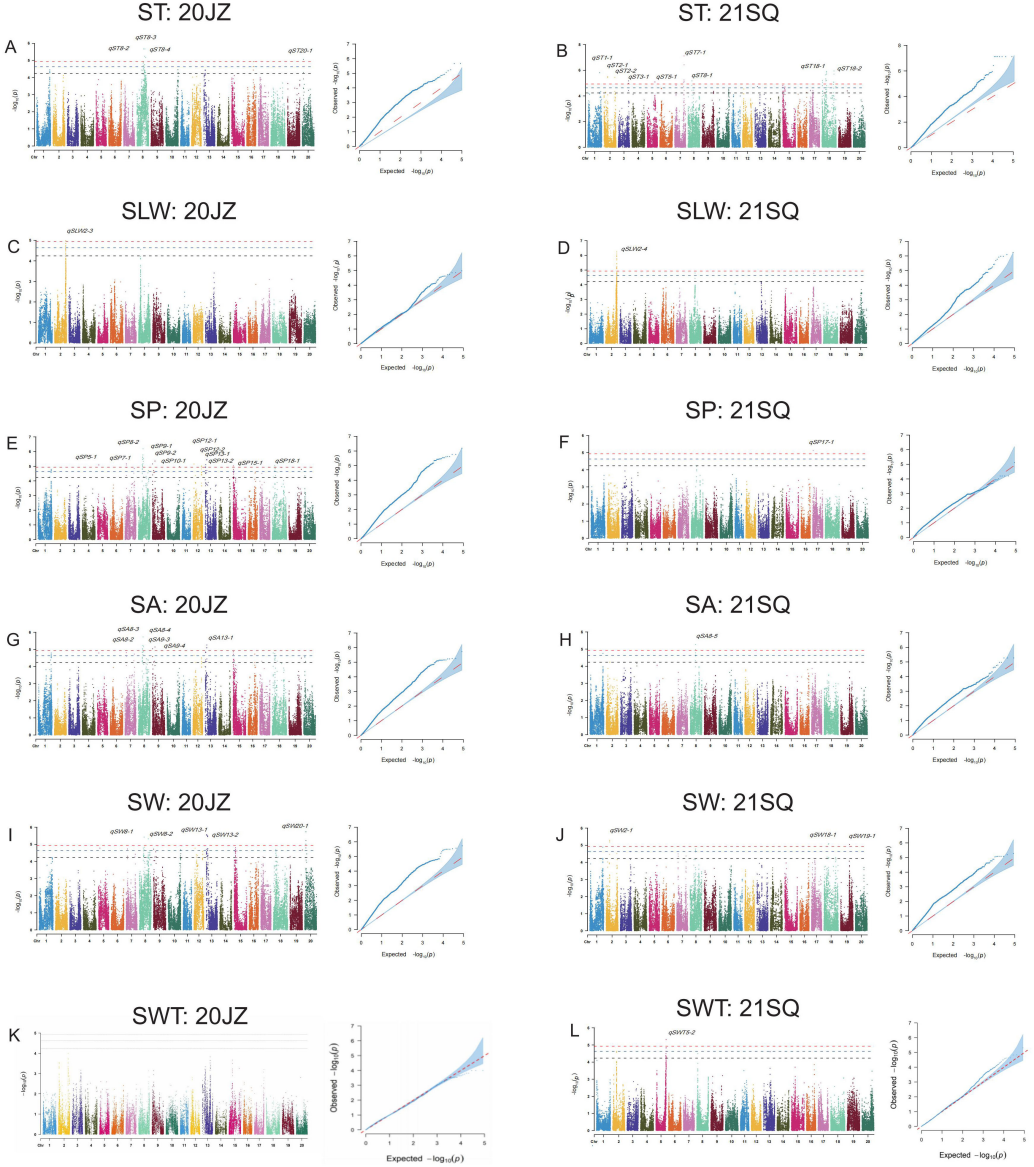
Manhattan plots and quantile-quantile (QQ) plots for **(A, B)** ST, seed thickness; **(C, D)** SLW, seed length to width; **(E, F)** SP, seed perimeter; **(G, H)** SA, seed area; **(I, J)** SW, seed width; **(K, L)** SWT, seed width to seed thickness using TASSEL under two environments (20JZ and 21SQ).

In this study, we employed ICIM for linkage analysis and EMMAX and TASSEL for genome-wide association studies to identify quantitative trait loci (QTL) regulating seed shape. A total of 98 QTL corresponding to 68 unique QTL loci were identified by all three methods (**Table 2**; **Appendixes D, E, F, G**). Linkage mapping with ICIM detected a total of 44 QTL associated with seed-related traits (**Figure 3**; **Appendixes D**). The number of QTL and their collectively explained phenotypic variation for each trait were as follows: eight for SL (29.67%), five for SW (26.59%), nine for ST (43.76%), seven for SP (29.66%), seven for SA (36.83%), four for SLT (15.87%), two for SLW (8.44%), and two for SWT (7.21%). Through EMMAX analysis, a total of 10 seed shape-associated QTL were identified on chromosomes 2, 5, 6, and 8, including four QTL located on each of chromosomes 2 and 8 (**Figure 4**; **Appendix E**). TASSEL-based mapping detected 44 QTL, which were predominantly distributed on chromosomes 2, 8, 9, 13, and 18, with 5, 11, 4, 5, and 4 QTL identified on these chromosomes, respectively (**Figure 5**; **Appendixes F**, I).

**Table 2:** Statistic of QTL identified by different methods.

Traits	20JZ	21SQ	Total QTL	Chromosome	Total PVE (%)	Method
SL	2	6	8	2; 3; 4; 5; 8; 9; 11; 17	29.67	ICIM
SW	2	3	5	7; 10; 11; 17	26.59	ICIM
ST	5	4	9	3; 11; 14; 17; 20	43.76	ICIM
SP	3	4	7	4; 5; 9; 10; 11; 19; 20	29.66	ICIM
SA	3	4	7	9; 10; 11; 20	36.83	ICIM
SLT	1	3	4	5; 6; 11	15.87	ICIM
SLW	1	1	2	6	8.44	ICIM
SWT	0	2	2	5; 6	7.21	ICIM
SL	0	0	0	NA	NA	EMMAX
SW	0	0	0	NA	NA	EMMAX
ST	1	0	1	6	NA	EMMAX
SP	0	1	1	8	NA	EMMAX
SA	0	1	1	8	NA	EMMAX
SLT	1	1	2	2; 8	NA	EMMAX
SLW	2	1	3	2; 8	NA	EMMAX
SWT	0	2	2	2; 5	NA	EMMAX
SL	0	0	0	NA	NA	TASSEL
SW	5	3	8	2; 8; 13; 18; 19; 20	62.46	TASSEL
ST	4	9	13	1; 2; 3; 5; 7; 8; 18; 20	92.44	TASSEL
SP	12	1	13	5; 7; 8; 9; 10; 12; 13; 15; 17; 18	97.69	TASSEL
SA	6	1	7	8; 9; 13	50.21	TASSEL
SLT	0	0	0	NA	NA	TASSEL
SLW	1	1	2	2	17.3	TASSEL
SWT	0	1	1	5	7.52	TASSEL

Among these identified QTL, 21 genomic regions overlapped with 39 QTL detected by multiple mapping methods or for different traits. These overlapping QTL are considered robust, stable, and highly confident, as their repeated identification underscores their reliability (**Appendix G**). Notably, the locus *qSLW2-1* (Chr02: 43,973,626–46,364,051) was co-localized with several other QTL—*qSLT2-1*, *qST2-2*, *qSLW2-2*, *qSLW2-3*, and *qSLW2-4*—using both mapping methods and across two environments (20JZ and 21SQ). Among these, *qSLW2–4* displayed the highest phenotypic variance explained (PVE) value of 9.83%. Furthermore, *qSWT5-1* (Chr05: 40,434,511–42,218,304) was consistently detected by both methods in the 21SQ environment, explaining 7.52% of the phenotypic variation in SWT; this locus was designated *qSWT5-2*. Similarly, *qSA8-1* (Chr08: 35,457,204–36,135,707) overlapped with *qSP8-1*, *qSW8-2*, and *qST8–4* across analytical methods and environments. Of these, *qSW8–2* exhibited the highest PVE value, at 8.01% (**Appendixes D, E, F, G**).

### 3.4 Candidate gene mining

**Figure 6:**
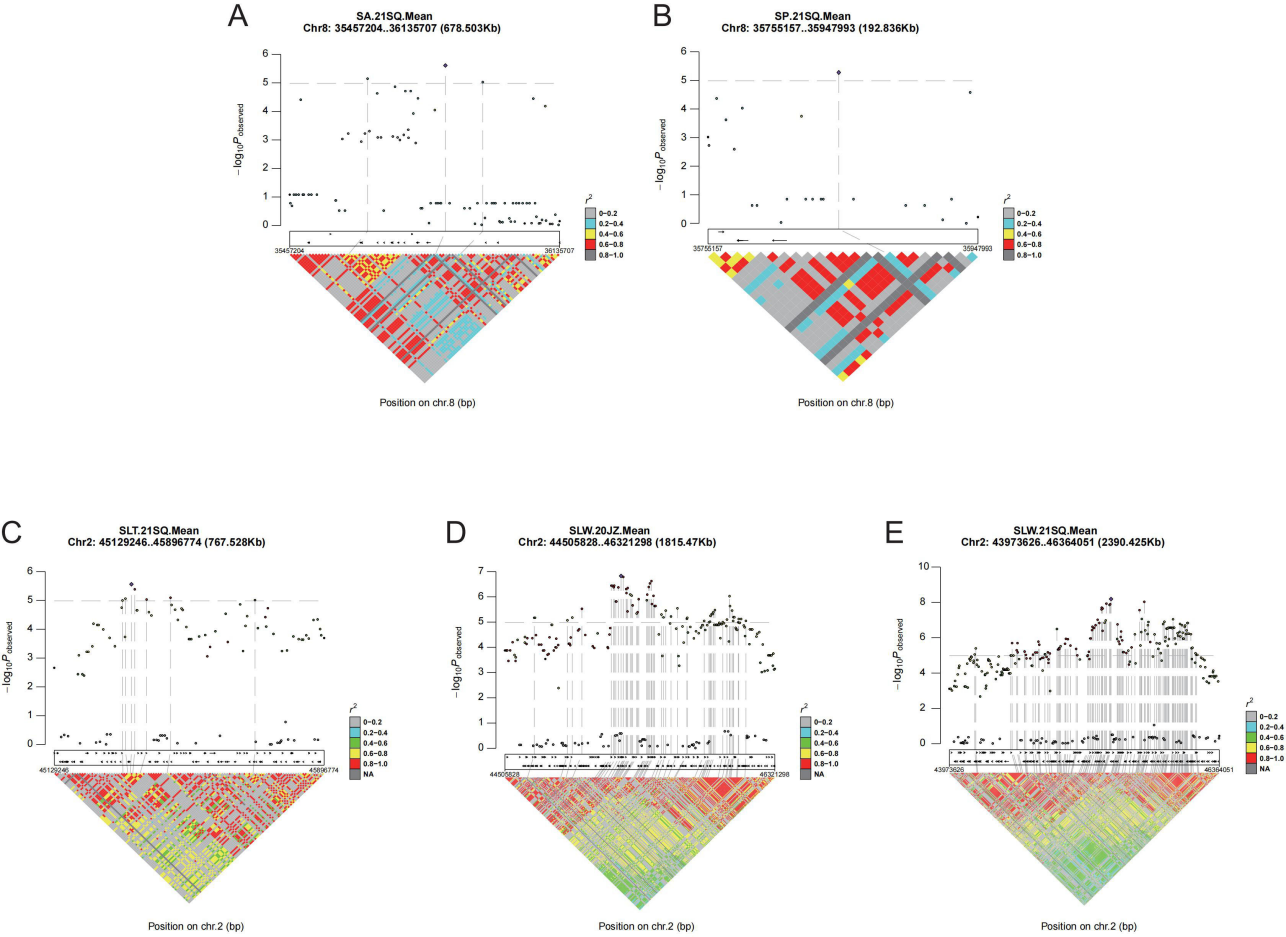
Linkage disequilibrium analysis of associated regions. **(A–E)** integrated map of association and linkage disequilibrium of *qSA8-1, qSP8-1, qSLT2-1, qSLW2-2*, and *qSLW2-1*, respectively.

*qSWT5-1* and *qSA8–1* were consistently detected by both approaches and exhibited high PVE. Notably, within the *qSWT5–1* interval (Chr05:40,434,511–42,218,304), the previously cloned gene *GmST05* (Chr05:41,852,863–41,854,961) (Duan et al., 2022) was physically located. Linkage disequilibrium (LD) analysis of *qSLW2–1* revealed an LD block spanning Chr02:43,973,626–46,363,056 encompassing 285 genes. For *qSA8-1*, one LD block (Chr08:35,652,893–35,848,556) was identified to contain 11 genes (**Figure 6**; **Appendixes E**).

**Figure 7:**
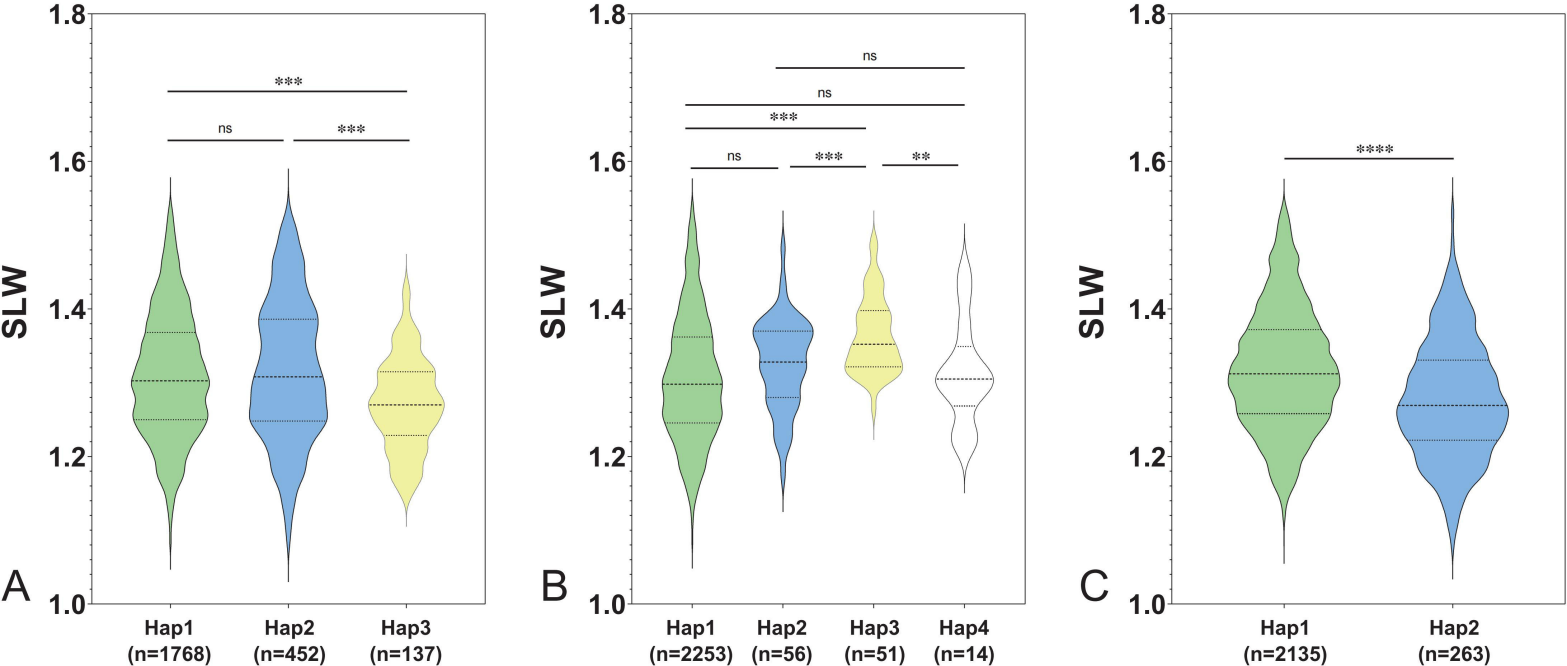
Phenotypic comparison among haplotypes of candidate genes. **(A–C)** represent the phenotype differences in SLW among haplotypes of *Glyma.02G255900, Glyma.02G270100*, and *Glyma.02G275200*, respectively. ns:not significant (*P*≥0.05); **:*p*<0.01; ***:*p*<0.001; ****:*p*<0.0001.

SNP variation analysis revealed that, among the aforementioned 296 genes, 291 contained SNPs classified as nonsynonymous, stop-gain, stop-loss (loss of stop codon), or alternative splicing mutations (**Appendix H**). Gene expression analysis showed that 12 of those 291 genes were seed-specific expressed (**Appendix I**). Based on the phenotypic and genotypic data of the NAM population, haplotypes of 291 genes were analyzed. A total of 682 haplotypes were inferred for 217 genes, of which 154 genes showed significant differences between haplotypes (**Appendix J**). Taken together, three genes exclusively expressed in seeds, namely *Glyma.02G255900*, *Glyma.02G270100*, and *Glyma.02G275200* showed significant phenotypic differences between haplotypes as well (**Figure 7**; **Appendixes I, K**), which were functionally characterized as nucleolar histone methyltransferase-related protein, FAM91A1, and early nodulin-like protein 20, respectively, and were therefore selected as candidate genes for seed shape regulation.

### 3.5 Genomic selection of seed shape related traits

**Figure 8:**
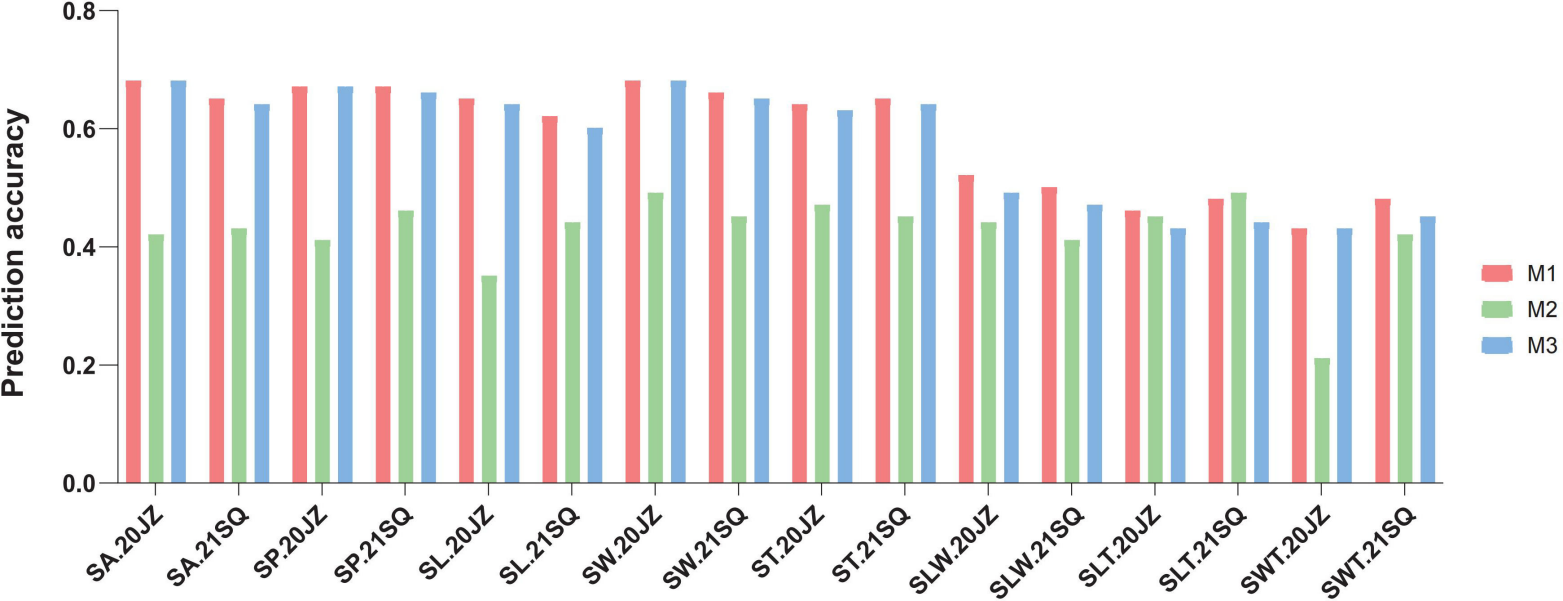
Genomics selection of different seed shape-related traits. M1, Genome wide SNP marker; M2, trait associated SNP markers; M3, Genome wide SNP marker.

To construct the GS model for seed-related traits, three different marker sets, namely genome-wide markers (M1), trait-associated markers (M2), and genome-wide markers excluding trait-associated markers (M3), were used. Results showed that the trait-associated marker set (M2) showed relatively lower prediction accuracy compared to genome-wide markers (M1 and M3). For the measured seed shape traits (SL, SW, ST, SP, and SA), the prediction accuracy by genome-wide markers reached 0.60–0.68. This represents a 40.2% to 60.8% increase over the accuracy achieved using the trait-associated marker set M2 (0.35–0.49). Minimal differences in accuracy were observed between the genome-wide markers and sets M1 and M3 (**Figure 8**; **Appendix L**). The derived traits (SLT, SLW, and SWT) exhibited lower prediction accuracy, ranging from 0.41 to 0.52, in contrast to the higher accuracy achieved for the directly measured traits. The prediction accuracy of genomic selection showed no discernible pattern across the different environments.

## 4 Discussion

In this study, seed shape was characterized by eight related traits, namely SL, SW, ST, SP, SA, SLT, SLW, and SWT, of which SLT, SLW, and SWT are derived traits obtained by division among SL, SW, and ST. Of the five measured traits, all exhibited high correlations with each other (0.61–0.98) (**Figures 2A, B**), clustered together and distinctly separated from derived traits in the plant trait network (**Figure 2C**). SA and SP are also derived from seed length and width through calculation. In this context, SL, SW, and ST are the key components determining seed size and shape. Correlation analysis revealed that SL exhibited the weakest correlation with HSW among the three seed shape-related traits in different environments (20JZ and 21SQ) (**Figures 2A, B**). Meanwhile, path analysis indicated that all measured traits (SL, SW, ST, SP, and SA) had positive total effects on hundred-seed weight, while SW was the only trait exhibiting a positive direct effect (**Figure 2D**; **Appendix C**), suggesting its decisive role in determining soybean HSW in this study.

However, this pattern is not universally consistent. Multiple studies have reported significant correlations between seed thickness (ST) and HSW under varying environmental conditions. For instance, in a recombinant inbred line (RIL) population developed from a cross between Dongnong46 and L−100, HSW demonstrated the strongest association with ST across three environments (18XY, 18AC, and 18HL), whereas it correlated most strongly with SW in the 17XY environment (Teng et al., 2019). Similarly, in a RIL population derived from Guizao1 and B13, the highest regression coefficient (R²) was observed between HSW and SW in five environments, while the strongest relationship with ST was detected in one environment (Luo et al., 2023). Another study using a RIL population from the cross “Zheng92116” × “Qihuang30” found that HSW showed the highest correlation with SW in two environments, with ST in one environment, and exhibited equal correlation strength with both SW and ST in an additional environment (Sun et al., 2021). It has been reported that soybean seed shape and size have undergone domestication selection, and the importance of ST for hundred-seed weight progressively increases from wild soybeans to landraces and improved cultivars (Niu et al., 2012). Collectively, SW and ST may be the key phenotypic determinants influencing HSW in soybean.

Fifty-seven loci overlapped with previous studies, accounting for 58.16% of the total QTL, while 41 loci were novel (**Appendix M**). As the seed shape-related traits varied in different environments with broad-sense heritability over 0.60, genetic effect on the seed shape related traits is probably higher than environmental effects. A number of regulatory genes controlling soybean seed shape have already been cloned, with major efforts focused on transcriptional regulation, phytohormone signaling and homeostasis, and metabolic pathways (Zhang et al., 2024). In this study, three seed-specific genes with significant phenotypic differences between haplotypes were selected as candidate genes regulating seed shape. *Glyma.02G255900* encodes a nucleolar histone methyltransferase-related protein and might influence the global methylation status of genes in seeds. *Glyma.02G270100* was annotated as a FAM91A1 protein. The homologue of which (*LTD1*) in rice located on the plasma membrane, endoplasmic reticulum, and multivesicular bodies, could interact with TFB2, and CycH1;1 and is involved in cell cycle regulation. Knockdown of LTD1 resulted in reduced tiller number, grain number per plant, and as well as 1,000-grain weight (Yang et al., 2025b). *Glyma.02G270100* might be involved in HSW and seed shape regulation via a similar mechanism in soybean; however, further investigation is needed. *Glyma.02G275200* encodes a nodulin-like protein 20. Although nodulin-like proteins were presumed to function in the nodulation process, recent studies also suggest that early nodulin-like (ENODL) genes may play a vital role in the formation and development of underground storage organs in plants (Zhao et al., 2023). Seed shape is formed during seed development, which could be possibly regulated by early nodulin-like genes as well.

This study identified 59 unique QTLs related to seed shape‑related traits, further confirming that seed morphology is a complex quantitative trait. Because the broad‑sense heritability of seed shape‑related traits was relatively high (0.67–0.90), genomic selection analysis could be performed. Using marker set M2 (trait‑associated markers), the prediction accuracy for seed shape‑related traits ranged from 0.43 to 0.48. Notably, the M1 marker set, which contained an additional 80,212 SNPs not associated with seed shape, increased prediction accuracy by 36.2%–85.7%. These findings are inconsistent with earlier research on soybean branching and stem termination traits (Wang et al., 2024; Yang et al., 2025a). However, the results of genomic selection (GS) and marker‑assisted selection (MAS) for predicting soybean hundred‑seed weight showed that the average prediction accuracy of GS was superior to that of MAS, which is consistent with the results of this study (Zhang et al., 2016). This discrepancy may be due to incomplete identification of seed shape‑associated markers in the current study, where undiscovered markers and missing heritability might play an important role in shaping prediction outcomes.

## 5 Conclusion

This study conducted both phenotypic and genetic analyses of seed shape-related traits in a RIL population. The broad-sense heritability of different traits ranged from 0.67 to 0.90, with SW ranking the highest. The interrelationship analysis between HSW and seed shape-related traits revealed that ST and SW might be key traits contributing to HSW. A total of 98 QTL were mapped with 60 QTL co-localized and corresponding to 59 unique QTL regions. Three genes, *Glyma.02G255900, Glyma.02G270100*, and *Glyma.02G275200*, were identified as candidates for seed shape regulation, which are under investigation. Genomic prediction accuracy of seed shape-related traits ranged from 0.62 to 0.68, making the application of genomic selection feasible in the breeding program.

## Data Availability

The data presented in the study are deposited in the Soybean Functional Genomics and Breeding repository, direct access to data through link https://sfgb.rmbreeding.cn/about/data.

## Appendix A

The ANOVA analysis of seed shape related traits and the proportion of variance explained by genotype.

## Appendix B

PTN analysis of seed shape related traits and HSW.

## Appendix C

Path analysis of seed shape related traits and HSW.

## Appendix D

Seed traits related QTL mapped by ICIM in two different environments.

## Appendix E

Seed traits related QTL mapped by EMMAX in two different environments.

## Appendix F

Seed shape related QTL identified by TASSEL.

## Appendix G

The overlapping QTL detected by either multiple mapping methods or for different traits.

## Appendix H

SNP variation related to genes in *qSLW2-1* and *qSA8-1*.

## Appendix I

Expression profile of genes within cadidate region among different tissues.

## Appendix J

Haplotyoe sequence of genes within *qSLW2-1* and *qSA8-1*.

## Appendix K

Statitistic significance between haplotypes of genes within *qSLW2-1* and *qSA8-1* by Wilconxon.

## Appendix L

GS prediction accuracy of soybean seed shape related traits based on different marker sets using RRBLUP.

## Appendix M

Seed trait related QTL identified overlapped with references.
